# Extraoral Osseous Choristoma in the Head and Neck Region: Case Report and Literature Review

**DOI:** 10.1155/2019/8532356

**Published:** 2019-05-28

**Authors:** Philipp Arens, Andrea Ullrich, Heidi Olze, Florian Cornelius Uecker

**Affiliations:** ^1^Charité—Universitätsmedizin Berlin, Corporate Member of Freie Universität Berlin, Humboldt-Universität zu Berlin, and Berlin Institute of Health, Department of Otorhinolaryngology, Charitéplatz 1, 10117 Berlin, Germany; ^2^Charité—Universitätsmedizin Berlin, Corporate Member of Freie Universität Berlin, Humboldt-Universität zu Berlin, and Berlin Institute of Health, Department of Pathology, Charitéplatz 1, 10117 Berlin, Germany

## Abstract

An osseous choristoma is a benign tumor consisting of regular bone tissue in an irregular localization. Choristomas in the head and neck region are rare. Most frequently, they are found in the region of the tongue or oral mucosa. There are also very few reports on osseous choristomas in the submandibular region. We present the case of a woman with a large, caudal osseous choristoma within the lateral cervical triangle. Literature review is given about all of the reported cases in the region of the neck. The pathogenesis is yet unexplained. Our case supports the theory that the development of an osseous choristoma is a reaction to a former trauma. Cervical osseous choristomas are seldom, but they represent an important differential diagnosis when dealing with a cervical tumor.

## 1. Introduction

An osseous choristoma is a benign tumor consisting of regular bone tissue in an irregular localization [1]. Choristomas are most frequently found in the region of the tongue or oral mucosa [2]. Beyond these localizations, choristomas in the head and neck region are rare. We report on a case involving a large, caudal osseous choristoma within the lateral cervical triangle. According to our research in the region of the cervical soft tissue, only four osseous choristomas have been described in the English-language literature to date. These choristomas all presented within the craniocervical soft tissue of the submental or submandibular region [3–7].

## 2. Case Report

**Figure 1:**
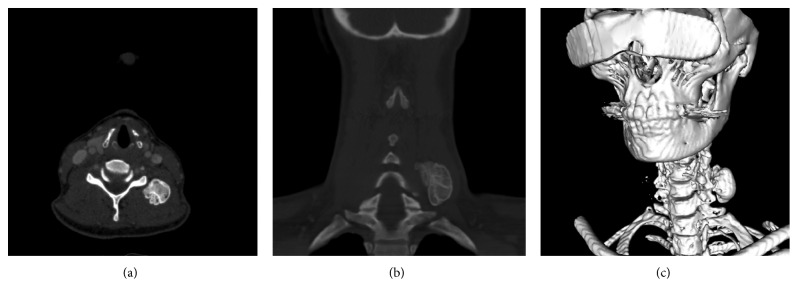
Preoperative computertomography scan: axial (a), coronary (b), and 3D-reconstruction (c). The CT scan shows a calcificated mass without contact to the skeleton.

**Figure 2:**
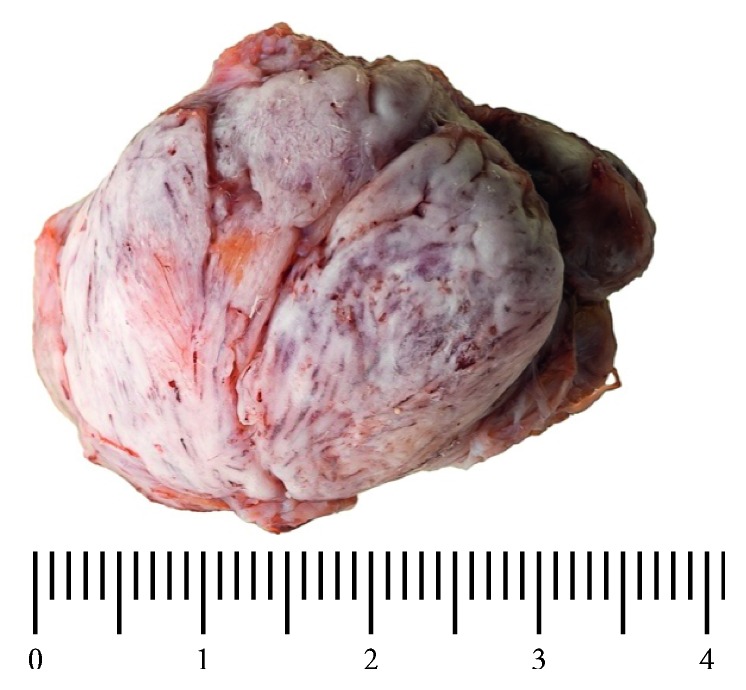
Display of the removed formalin-fixated specimen (37 × 22 × 22 mm, 12 g, presenting as a round, bony mass smoothly covered by a narrow lamella of connective tissue).

**Figure 3:**
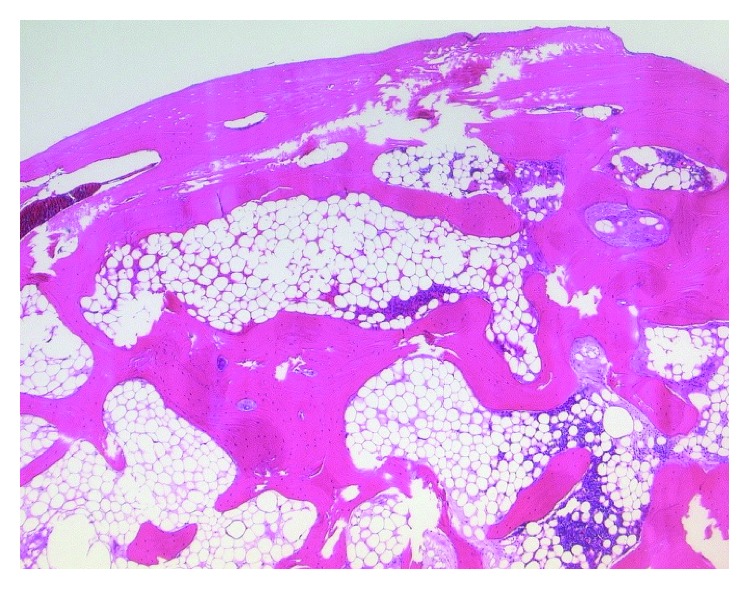
Histopathological appearance of the lesion (haematoxylin and eosin staining, magnification 10x). Beneath the surrounding compact bone, the structure consists of cancellous bone tissue with regular medullary cavities.

Our report refers to a 46-year-old female patient who presented to our hospital. She had noticed a firm, space-occupying lesion in the left cervical soft tissue that had been increased in size slowly over a period of several months. According to the patient, a cervical lymph node biopsy had been performed in the same localization 12 years ago. Apart from a nonspecific inflammation, the course had been inconspicuous. In the clinical examination, the cervical mass was palpable. It felt firm and could be moved independently of the skin, but not independently of the cervical soft tissue. Ultrasound revealed a solid structure with complete dorsal acoustic attenuation. Computer tomography of the cervical soft tissue showed a solid structure measuring approx. 24 × 21 × 33 mm, which seemed to be consistent with a calcification and which had no contact to adjacent bony structures (see Figure 1). Intraoperatively, a hard, bony, smoothly covered mass with a largest diameter of approximately 4 cm was completely extirpated, with primary closure of the wound. Postoperative healing was free of complications. The formalin-fixated specimen had size of 37 × 22 × 22 mm, and the weight was 12 g (see Figure 2). Histopathology of the specimen processed with a haematoxylin and eosin staining revealed a round, bony mass smoothly covered by a narrow lamella of connective tissue. Beneath the surrounding compact bone, the structure consisted of cancellous bone tissue with regular medullary cavities enclosing yellow marrow, as well as differently sized areas of mature hematopoietic bone marrow, suggesting an ectopic formation of regularly differentiated bone tissue (see Figure 3).

## 3. Discussion

The term “osseous choristoma” and its definition can be attributed to Krolls et al. They described several cases of ectopic bone tissue in the region of the oral soft tissue [1]. Generally speaking, choristomas are rare. In the head and neck region, they are predominantly found within the tongue and the surrounding soft tissue [2]. Clinical presentations of osseous choristomas usually take form of painless, slowly progressive space-occupying lesions. Infections are seldom. As choristomas increase in size, functional complaints, such as dysphagia, emerge [2]. In the neck region, the number of reported cases is extremely low (see Table 1) [3–7]. Psimopoulou and Antoniades described one case of a submental choristoma. Johann et al., Kamburoğlu et al., and Shimada et al. have each described one case of submandibular osseous choristoma. In the German-language literature, Schmal et al. reported on a case in the region of the mandibular angle. In the course of our literature research, we did not encounter a single published case of an osseous choristoma in the caudal region of the lateral cervical triangle. Within the region of the tongue and oral cavity, most cases occur in women [2]. The synopsis of the few published cases in the region of the cervical soft parts shows a deviating tendency. In this localization, osseous choristomas seem to occur with the same frequency in men and women. The mean age is 45.33 ± 10.16 years. However, due to the small number of cases, reliable statements regarding mean age and distribution are not possible.

There are various clinical differential diagnoses of head and neck masses at the caudal region of the lateral cervical triangle. In knowledge of the computertomographic findings, the amount of differential diagnoses is reduced to bony or calcificated lesions, such as myositis ossificans, calcified lymph nodes, calcified hemangioma, or osseous choristoma. Calcified lymph nodes are associated with tuberculosis, metastatic thyroid carcinomas, healed necrotic abscesses, or non-Hodgkin lymphoma [8]. Knowing the histopathological appearance of our specimen, these differential diagnoses were quickly ruled out, due to the fact that in addition to the regular structured cancellous bone tissue, bone marrow tissue was found.

The pathogenesis of osseous choristoma is yet unexplained. The literature does not describe an increased risk of malignant transformation. Several theories about the development of these lesions exist. As potential pathomechanisms, a hereditary malformation and a reaction to a previous trauma are discussed [9]. The latter hypothesis is supported by the case we report here. Our patient's anamnesis revealed a close correlation to a previous intervention in the neck region. She reported that a cervical lymph node biopsy had been performed in the same location years before. Unfortunately, it was not possible for us to acquire the old histopathological report, so that the correlation between these two incidents remains unclear.

Treatment of choristomas involves surgical extirpation of the lesion. Recurrences are seldom, yet reported in the literature, meaning that follow-up examinations can nevertheless be beneficial [10].

## 4. Conclusion

Cervical osseous choristomas are seldom, but they represent an important differential diagnosis to myositis ossificans and especially to calcifying cervical lymph nodes of different causes; therefore, they are of broader clinical interest.

## Figures and Tables

**Table 1:** Overview of the cases of osseous choristoma in the cervical soft tissues published in the literature. The table shows that all of the previous described lesions have been found in the submandibular or submental region.

	Sex	Age (years)	Localization
Kamburoğlu et al. [5]	w	33	Submandibular
Shimada et al. [3]	m	50	Submandibular
Johann et al. [4]	m	32	Submandibular
Psimopoulou and Antoniades [6]	w	50	Submental
Schmal et al. [7]	m	61	Mandibular angle
